# Phytochemicals as Regulators of Tumor Glycolysis and Hypoxia Signaling Pathways: Evidence from In Vitro Studies

**DOI:** 10.3390/ph15070808

**Published:** 2022-06-28

**Authors:** Ioana-Ecaterina Pralea, Alina-Maria Petrache, Adrian Bogdan Tigu, Diana Gulei, Radu-Cristian Moldovan, Maria Ilieș, Raul Nicoară, Simona-Codruța Hegheș, Alina Uifălean, Cristina-Adela Iuga

**Affiliations:** 1 MEDFUTURE Research Center for Advanced Medicine, Department of Proteomics and Metabolomics, “Iuliu Hațieganu” University of Medicine and Pharmacy, Louis Pasteur Street 4-6, 400349 Cluj-Napoca, Romania; pralea.ioana@umfcluj.ro (I.-E.P.); alina.mornea@gmail.com (A.-M.P.); moldovan.radu@umfcluj.ro (R.-C.M.); ilies.maria@umfcluj.ro (M.I.); nicoara.raul@gmail.com (R.N.); iugac@umfcluj.ro (C.-A.I.); 2 Department of Pharmaceutical Biochemistry and Clinical Laboratory, Faculty of Pharmacy, “Iuliu Hațieganu” University of Medicine and Pharmacy Cluj-Napoca, Louis Pasteur Street 6, 400349 Cluj-Napoca, Romania; 3 MEDFUTURE Research Center for Advanced Medicine, Department of Translational Medicine, “Iuliu Hațieganu” University of Medicine and Pharmacy, Louis Pasteur Street 4-6, 400349 Cluj-Napoca, Romania; adrianbogdantigu@gmail.com; 4 MEDFUTURE Research Center for Advanced Medicine, Department of In Vivo Studies-Animal Facility, “Iuliu Hațieganu” University of Medicine and Pharmacy, Louis Pasteur Street 4-6, 400349 Cluj-Napoca, Romania; diana.gulei@umfcluj.ro; 5 Department of Pharmaceutical Analysis, Faculty of Pharmacy, “Iuliu Hațieganu” University of Medicine and Pharmacy, Louis Pasteur Street 6, 400349 Cluj-Napoca, Romania; cmaier@umfcluj.ro

**Keywords:** phytochemicals, tumor glycolysis, tumor hypoxia, Warburg effect, HIF-1 signaling pathway, metabolomics

## Abstract

The full understanding of the complex nature of cancer still faces many challenges, as cancers arise not as a result of a single target disruption but rather involving successive genetic and epigenetic alterations leading to multiple altered metabolic pathways. In this light, the need for a multitargeted, safe and effective therapy becomes essential. Substantial experimental evidence upholds the potential of plant-derived compounds to interfere in several important pathways, such as tumor glycolysis and the upstream regulating mechanisms of hypoxia. Herein, we present a comprehensive overview of the natural compounds which demonstrated, in vitro studies, an effective anticancer activity by affecting key regulators of the glycolytic pathway such as glucose transporters, hexokinases, phosphofructokinase, pyruvate kinase or lactate dehydrogenase. Moreover, we assessed how phytochemicals could interfere in HIF-1 synthesis, stabilization, accumulation, and transactivation, emphasizing PI3K/Akt/mTOR and MAPK/ERK pathways as important signaling cascades in HIF-1 activation. Special consideration was given to cell culture-based metabolomics as one of the most sensitive, accurate, and comprising approaches for understanding the response of cancer cell metabolome to phytochemicals.

## 1. Introduction

The reliance on aerobic glycolysis as the main energy-generating pathway is a signature of most cancerous cells. Besides ATP, the accelerated glycolysis rate can provide valuable building blocks required for DNA, protein, and lipid synthesis to sustain the intensive tumor proliferation. The shift to a glycolytic metabolism from oxidative phosphorylation, known as the Warburg effect, takes place even in the presence of oxygen and has been shown to promote tumorigenesis and malignancy progression [1]. Therefore, tumor glycolysis constitutes an attractive target for cancer therapy. The glycolytic cascade can be controlled not only through the glucose membrane transporters but also through the rate-limiting enzymes, such as hexokinase 2 (HK2), phosphofructokinase 1 (PFK-1), and pyruvate kinase 2 (PKM2). These key control points can be regulated through direct interaction, or, more often, their expression is controlled at the transcriptional level by the hypoxia-inducible factor 1 (HIF-1). In fact, HIF-1 is responsible for the transcriptional activation of over 100 downstream genes, which regulate different processes necessary for tumor growth and development [2]. Overexpression of the oxygen-dependent HIF-1α subunit has been associated with angiogenesis, metastasis, and cell survival. To regulate HIF-1α expression, one possibility is through growth factors signaling pathways, such as the phosphoinositide 3-kinase/protein kinase B/mammalian target of rapamycin (PI3K/Akt/mTOR) signaling pathway or mitogen-activated protein kinase/extracellular signal-regulated kinase (MAPK/ERK) pathway.

From the last key enzyme of the glycolytic pathway to the first upstream regulators of HIF-1α, there are multiple control points where the pro-survival mechanisms can be hindered. One of the advantages of using natural compounds as anticancer agents is their multistep targeting potential, interfering with several checkpoints of the carcinogenic cascade. As an example, apigenin, an abundantly present flavonoid, was shown to suppress the expression of glucose transporter 1 (GLUT1) in prostate cancer cells [3] and adenoid cystic carcinoma cells [4], of HK2 in hepatocarcinoma cells [5] and in osteosarcoma cells [6], of PKM2 in colorectal cancer cells [7]. Moreover, the flavonoid can also interfere in the upstream regulatory pathways, by reducing HIF-1α stability [8] or inhibiting the PI3K/Akt/mTOR signaling pathway, causing apoptosis and autophagy [9]. In addition to the multifold identified mechanisms, apigenin displays an excellent safety profile, even at high doses [10,11].

Given the limited efficacy of single-target drugs against complex biochemical diseases, such as cancers, and the need for safe anticancer agents, natural compounds with antitumor effects hold tremendous potential. Therefore, this paper aims to present a comprehensive overview of the natural compounds which demonstrated, in in vitro studies, an effective anticancer activity by targeting the glycolytic pathway or the hypoxia signaling pathways, including PI3K/Akt/mTOR and MAPK/ERK. Special attention will be given to cell culture-based metabolomics due to its unquestionable advantages in terms of accuracy and sensitivity for assessing the phytochemicals impact on cancer cell metabolome. 

## 2. Phytochemicals Targeting Glycolysis and Hypoxia Signaling Pathways—Structural Classification and Natural Sources

In the last 40 years, approximately 25% of all approved anticancer drugs are derived from phytochemicals, and a few dozen others have been shown antitumor potential in in vitro and/or in vivo models [12]. In recent years, an increasing number of phytochemicals have been shown to impair tumor cell growth by targeting key regulators of glycolytic and hypoxia signaling pathways. Structurally, these phytochemicals can be classified into polyphenols, terpenoids, alkaloids, organosulphur compounds, phytosterols, and nitrogen-containing compounds [13]. Many of these compounds are found in various vegetables, fruits, grains, or spices, creating the opportunity to use components of our daily diet as effective chemopreventive agents [14].

**Figure 1:**
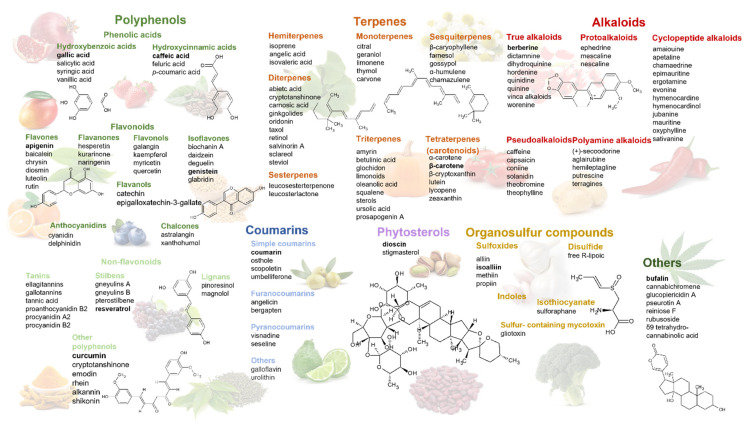
Phytochemicals targeting glycolysis and hypoxia signaling pathways: classification, main representants, and chemical structures (the chemical structure belongs to the compound marked in bold).

Polyphenols are compounds with phenolic structural features, naturally occurring in conjugated forms (glycosides), with one or more sugar residues and acylated sugars linked to the polyphenol skeleton. Depending on the number of phenol rings, the attached substituents and the linkage type, polyphenols can be classified into different groups and subgroups, such as phenolic acids, flavonoids, non-flavonoids, and other polyphenols (Figure 1). Natural sources of polyphenols include berries, grapes, nuts, or soy seeds. The antitumor activity of polyphenols arises from their long-established antioxidant and anti-inflammatory properties [15,16], along with newer identified mechanisms, such as regulators of key enzymes of glycolysis and the HIF-1 signaling pathway [17].

The most chemically and structurally diverse family of natural products, terpenoids, consist of five-carbon isoprene units assembled in various ways, with different functional groups and oxidized methyl groups. Depending on the carbon units, terpenoids are divided into monoterpenoids, diterpenoids, triterpenoids (free or conjugated), tetraterpenoids, and sesquiterpenoids. The best-known representative of the diterpenes is paclitaxel, whose antitumor activity was highlighted in 1962; today, it is being used in breast, lung, ovarian cancer, and Kaposi’s sarcoma treatment [12]. Important sources of terpenoids include carrots, tomatoes, pumpkins, and oranges.

Another important group of chemical compounds that serve as a rich source for drug discovery is represented by alkaloids, cyclic structures with at least one basic nitrogen atom. Most alkaloids are obtained from amino acids such as phenylalanine, tyrosine, tryptophan, ornithine, and lysine. From this heterogeneous group, the alkaloids from *Vinca rosea* (vincristine, vinblastine, vindesine, and vinorelbine) and those from *Berberis asiatica* (berberine) have stood out for their antitumor activity. The *Vinca* alkaloids are known for their microtubule-disruptive mechanisms, while berberine is due to its regulating cell cycle activity, along with antioxidant and anti-inflammatory properties [18]. Alkaloids occur abundantly in many plant species, including *Apocynaceae*, *Asteraceae*, *Loganiaceae*, *Rutaceae*, and *Papaveraceae* [19].

Organosulfur compounds, phytochemicals containing one or more sulfur atoms, were also claimed to own anticancer effects through several mechanisms. These include cell cycle arrest, apoptosis, inhibition of cell differentiation, and antioxidant defense [20]. Moreover, diallyl trisulfides and sulforaphane were shown cause cell death by interfering with HIF-1α-mediated signaling [21]. Plants containing organosulfur compounds mainly derive from two families, *Amaryllidaceae*, containing S-alk(en)yl-l-cysteine sulfoxides, and *Brassicaceae* containing S-methyl cysteine-l-sulfoxides. Therefore, the main sources of organosulfur compounds include garlic, onion, shallot, cabbage, and cauliflower [22].

Other phytochemicals, such as phytosterols [23], cardiotonic steroids [24], and cannabinoid derivates [25], have been experimentally demonstrated to pose antitumor effects. Their antitumor efficacy is largely dependent on the cancer type and the drug concentration, so understanding how exactly these molecules can regulate different tumoral processes is yet to be accomplished.

A thorough classification of the most important phytochemicals which regulate tumor glycolysis and hypoxia signaling, further presented in this paper, along with other class members, is presented in Figure 1.

## 3. Cell Culture-Based Metabolomics to Investigate Plant-Induced Metabolic Changes

There are multiple techniques and approaches which enable the identification, with different levels of accuracy, of the altered mechanisms and metabolic pathways caused by phytochemicals. Most studies have used glucose uptake assay, O_2_ consumption measurements, lactate production assays, ATP or NADPH/NADP measurements, or HK, PFK, PK, and LDH activity assays. In addition, the results were confirmed using western blot for the protein expression and real-time PCR for the mRNA level of the identified targets. Although these techniques are relatively easy to implement and are not very time-consuming, they do not capture the connections between all metabolites at a certain time. A very useful strategy in this regard would be represented by cell culture-based metabolomics. 

Metabolomics aims to provide a global assessment of the low-molecular-mass compounds present in a biological system, closely reflecting the phenotype of a cell, tissue, or organism. For cancer research, cell culture-based metabolomics provided significant evidence in understanding cancer development and predicting the response to treatment. Metabolomic applications based on cell cultures present several advantages such as easily controlled experimental variables, great reproducibility, reduced costs, and facile data interpretation.

Depending on the experimental purpose, two analytical strategies can be considered: targeted and untargeted approaches, both involving specific experimental methodologies. While untargeted metabolomics allows a broad identification and a relative quantification of all the measurable analytes in a sample, in targeted metabolomics, absolute quantification is performed on a specific, predefined group of metabolites.

For both approaches, detailed sample preparation strategies have been reviewed over the last decade [26,27]. In brief, compared to the usual sample preparation for biological fluids, sample preparation for cell culture samples requires harder processing methods, such as metabolism quenching and metabolite extraction. Quenching of the cellular activity represents a crucial step for both exo- and endometabolome analysis. According to Alvarez-Sanchez et al. [27], the quenching strategy should fulfill the following prerequisites: (i) fast inactivation of metabolism, (ii) preservation of sample integrity, (iii) should not induce significant variations in the concentration of metabolites, (iv) the sample should be amenable to subsequent steps of the analytical process. In the case of intracellular metabolites from suspension cells, the inhibition of enzyme activity can be performed by liquid nitrogen freezing, heating, and/or addition of cold solvents (buffered methanol, saline solution 0.9%), whereas for adherent cells, the optimal approach is considered liquid nitrogen freezing.

Metabolite extraction is closely dependent on the analysis strategy (targeted vs. profile), the biological sample type, and the metabolites’ physicochemical properties. In general, proteins and salts are considered potential interferents; therefore, they should be removed during the extraction step. For metabolite extractions, the quenching solution may also serve as an extractant, dependent on the protocol. Otherwise, an appropriate extraction strategy must be implemented, such as liquid-liquid extraction or solid-phase extraction.

Once the metabolites are extracted, the following step implies high throughput analysis, using nuclear magnetic resonance spectroscopy (NMR) and/or mass spectrometry (MS), the two platforms which continue to dominate the field of metabolomics. Both technologies provide complementary advantages in terms of sensitivity and precision, a combined approach ensuring greater metabolome coverage and higher confidence and accuracy of metabolite identification and quantification. Next, the datasets generated from NMR or MS are subjected to data processing and, finally, to data interpretation and integration, when results can be integrated with other ‘omics’ data.

One of the applications of cell culture metabolomics is deciphering the antitumoral molecular mechanisms of phytochemicals by exposing cancer cells to various doses of dietary compounds. The experimental design should consider several key aspects which must be taken into account: the objectives of the study (targeted/untargeted strategy, pathway reconstruction, enrichment analysis, biomarker identification), the availability of appropriate analytical equipment and standard operating procedures, the availability of various metabolite standards, in vitro models (primary cells, cell lines, spent culture media), the cell culture growth conditions, the tested phytochemicals (extracts or standard compounds), the physical and chemical properties of tested phytochemicals—solubility, stability, doses and exposure time, the selection of appropriate data analysis tools [28].

Clearly, metabolomics has its limitations compared with other methods applied for pathway identification. It requires a very fast sample preparation, a higher cell number for metabolite extraction, more expensive equipment, and higher analyst expertise. Still, its advantages in terms of accuracy, precision, sensitivity, and metabolite coverage make cell culture-based metabolomics a very valuable approach to identifying plant-induced metabolic changes. 

So far, most studies assessing the antitumoral metabolic mechanisms of phytochemicals were non-targeted, used polyphenolic compounds, and identified glycolytic pathway and glutathione metabolism as the main affected pathways (Table 1).

## 4. Aerobic Glycolysis as a Metabolic Rewiring to Sustain Cell Growth

One century ago, Otto Warburg hypothesized the idea that cancer cells are characterized by a metabolic injury due to the high degree of fermentation [38]. Since then, the reprogramed energy metabolism of cancer cells, known today as the Warburg effect, has become a cancer hallmark and a target for developing new therapeutic approaches [39]. The bioenergetic fingerprint of malignant cells is characterized by two biochemical events: high leaning on glucose uptake and aerobic glycolysis for energy production, even in normoxia, and optimal mitochondrial function [1,39].

In normal mammalian cells, lactic fermentation and aerobic respiration conclude in the production of energy. During lactic fermentation, glucose is processed to pyruvate via glycolysis and further reduced to lactate, usually exported into the bloodstream. The entire process yields 2 ATP molecules for each molecule of glucose. In the presence of oxygen, glucose is processed within three main pathways: glycolysis (cytosol), Krebs cycle (mitochondria), and oxidative phosphorylation (OXPHOS) (mitochondria) being completely oxidized to the end products, carbon dioxide, and water. The energetic efficiency of aerobic respiration is significantly higher (ca.15 times) compared to lactic fermentation [40].

Several proposals have been made regarding the advantages of the apparently inefficient energy metabolism of cancer cells. One hypothesis refers to the rapid ATP synthesis in aerobic glycolysis, where the rate of glucose transformation in lactate and subsequent ATP production is approximately 10–100 times higher than in the case of mitochondrial oxidation. Leveling the amount of ATP production in time shows that aerobic glycolysis compensates for the apparent inefficient ATP production through a faster metabolic rate, an advantage in a microenvironment with limited energy resources [41,42]. Another reason refers to the high proliferation and growth rate of malignant cells that require rapidly available carbon sources for anabolic processes. The excess carbon resulting from aerobic glycolysis is donated to multiple branching pathways to produce cellular building blocks: proteins, lipids, and nucleotides [43]. One such example consists of the de novo biosynthesis of serine through the phosphoglycerate dehydrogenase (PHGDH) enzyme [44]. Other proposals concern the acidification of the extracellular microenvironment through lactate secretion, affecting the tumor–stroma interface and allowing tumor invasion [45].

## 5. Natural Regulators of Aerobic Glycolysis

The glycolytic pathway can be controlled through different key molecules which regulate the glycolytic flux, such as glucose membrane transporters, hexokinase, phosphofructokinase, pyruvate kinase, or lactate dehydrogenase. Herein we review the compounds of natural origin which demonstrated, in in vitro studies, an effective anticancer activity by affecting these key glycolytic regulators. 

### 5.1. Inhibitors of Glucose Transporters (GLUTs)

GLUTs represent the main glucose transporters across the plasma membrane into the cytosol, a rate-limiting step in glucose metabolism. Of the 14 identified isoforms, GLUT1 is ubiquitously expressed and responsible for the basal glucose uptake in various cell types. GLUT1 overexpression was observed in many tumors, including squamous cell carcinoma, lung, pancreatic, hepatic, breast, ovarian, and colorectal cancers [46]. Alternately, GLUT3 isoform was found in tissues with high glucose demands (brain, placenta, testes) and its overexpression was reported in bladder cancer, hepatocellular carcinoma, non-small cell lung cancer, and glioblastoma [47]. Considered the insulin-responding glucose transporter, GLUT4 was primarily found in muscle cells and adipocytes. GLUTs expression is highly regulated by several oncogenes such as HIF-1 and c-Myc, or by p53 tumor suppressor protein [48].

Several flavonoids, such as genistein (Gen), phloretin, apigenin, and daidzein (Dai), were shown to regulate the expression of GLUT1 and GLUT4 in prostate cancer depending on the cell line phenotype. Hereof, Gen and Dai proved to be more efficient in reducing proliferation in androgen-sensitive LNCaP and PC-3-AR cells than in androgen-insensitive PC-3 or LNCaP-R cells. Apigenin and phloretin were the most efficient in reducing glucose uptake and modifying GLUT levels, leading to the highest antiproliferative effect [3]. In an extracellular and intracellular metabolomic study, Gen (11.04 µM and 22.44 µM) and Dai (36.39 µM and 52.24 µM) were also shown to decrease glucose uptake in MDA-MB-231 and MCF7 breast adenocarcinoma cells, regardless the estrogen expression status of the cells [33]. Another study investigated the capacity of Gen, myricetin, chrysin, resveratrol, kaempferol, and xanthohumol to block 2-deoxy-D-[1-3H] glucose (^3^H-DG) uptake in MCF7 breast adenocarcinoma cells. Of the listed polyphenols, kaempferol was the most potent inhibitor of ^3^H-DG uptake, with exposure of MCF7 cells at 30 mM for 24 h being associated with a 40% decrease in GLUT1 mRNA levels, leading to cytotoxic effects and proliferation arrest [49].

Silybin, a flavonoid extracted from *Silybum marianum*, was tested on doxorubicin-resistant colorectal cells with increased glucose dependency and high GLUT1 mRNA and protein expression. Silybin resulted in more cytotoxic in doxorubicin-resistant cells than in sensitive cells. Further, this flavonoid reduced glucose uptake by decreasing GLUT1 expression, as confirmed by western blot analysis [50]. In a similar experiment, silybin and its derivative, 2,3-dehydrosilybin, strongly inhibited glucose uptake in multiple cell lines, including adipocytes and Chinese hamster ovary cells, through direct competitive inhibition of GLUT4-mediated transport [51].

In addition, the effect of wogonin, a flavonoid extracted from *Scutellaria baicalensis Georgi,* was assessed in colorectal, ovarian, and hepatocellular cancer cells. The results indicated that wogonin suppressed glucose metabolism and cell proliferation in cancer cells expressing wild-type p53. More precisely, wogonin induced the phosphorylation and acetylation of p53, leading to stabilization and activation of wt-p53. In vitro and in vivo studies confirmed that wogonin can also regulate p53 downstream glycolytic factors, such as GLUT1, by decreasing its protein and mRNA level [52].

Another flavonoid, quercetin, inhibited glucose transport in L929 mouse fibroblast cells through a similar competitive mechanism. Using a flow cytometry assay, quercetin was shown to bind to an exofacial site of GLUT1 without being transported into cells [53]. In MCF7 and MDA-MB-231 breast adenocarcinoma cells, quercetin suppressed glucose uptake and lactic acid production, downregulating the expression of several glycolysis-related proteins, including GLUT1. Furthermore, in vivo and in vitro data suggested that quercetin’s ability to block cell glycolysis led to suppressed cell mobility via the Akt-mTOR pathway [54]. In the same cell lines, quercetin and epigallocatechin gallate (EGCG) inhibited ^3^H-DG uptake in a competitive and concentration-dependent manner by a mechanism unrelated to the presence of estrogen receptors [55]. Further studies confirmed the capacity of quercetin, as well as glabridin, an isoflavane found in the root extract of licorice, to block glucose uptake in MDA-MB-231 breast adenocarcinoma cells by downregulating the protein expression of GLUT1 [56]. 

Similarly, the capacity of EGCG to reduce GLUT1 expression was confirmed using another breast cancer cell line (4T1). In this study, glucose consumption was reduced by 30% and 35% following exposure to 160 μM and 240 μM EGCG, respectively, compared to control [57]. Also, the quercetin conjugated form seems to possess glucose inhibitory properties, as quercetin-3-O-glucoside was reported to reduce GLUT2 expression levels in Caco-2 colorectal adenocarcinoma cells [58]. Although quercetin acts as a glucose inhibitor in cancerous cells, a detailed in vitro/in vivo study showed that, in skeletal muscle cells, low concentrations of quercetin (0.1 nM and 1 nM) promote glucose uptake by increasing GLUT4 translocation via different signaling pathways. Therefore, for normal skeletal tissues, quercetin may maintain glucose homeostasis also underlying antidiabetic properties [59].

Similar dual effects were also observed for resveratrol, a non-flavonoid polyphenol. In different types of cancers, such as ovarian, prostate, breast, or bone, resveratrol was shown to inhibit glucose uptake, impairing cancer cell growth. Instead, in pathological conditions such as insulin resistance or diabetes, resveratrol increased glucose uptake and insulin sensitivity, favoring antidiabetic effects [46]. A comprehensive presentation of resveratrol’s inhibitory effects on glucose uptake was recently published by Zambrano et al. [46].

In an attempt to isolate bioactive compounds that inhibit cellular filopodia protrusion in the human epidermoid carcinoma A-431 cells, glucopiericidin A, a natural compound obtained from *Lechevalieria* sp. broth, was identified and tested. Metabolome analysis using capillary electrophoresis time-of-flight mass spectrometry (CE-TOFMS) in combination with [^13^C]-labeled glycolytic metabolites provided strong evidence that glucopiericidin A suppresses glycolysis by functionally targeting GLUT1-mediated glucose uptake [60].

Dhar et al. [61] exposed pancreatic cancer cell lines with different KRAS mutational statuses to bitter melon juice (derived from *Momordica charantia* fruits) and investigated the changes that occurred in the cellular metabolic profiles. The NMR spectra highlighted alterations in glycolysis and lactate pathways in the KRAS mutant cell line. The decrease in GLUT1 and monocarboxylate transporter 4 (MCT4) expressions were emphasized by immunofluorescence using confocal microscopy. The transporter’s expression was also affected in the wild-type KRAS cell line, although less pronounced.

### 5.2. Inhibitors of Sodium-Dependent Glucose Cotransporter (SGLT)

The second family transporter, SGLT, acts against the cell’s internal glucose concentration by applying an electrochemical sodium gradient. Of the five identified isoforms, SGLT1 is expressed in the intestine, lungs, brain, and liver, while SLGT2 is limited to the renal proximal tubule [62]. In the context of cancer, SGLT2 overexpression was found in prostate cancer, pancreatic and lung adenocarcinoma, glioblastoma, and breast cancer [62], and recently, investigators have also begun to study the potential of SGLT2 inhibitors in preclinical cancer studies [63].

Phlorizin, a naturally occurring flavonoid found in the bark of pear (*Pyrus communis*), represents the lead compound for synthetic analogs used in the treatment of type 2 diabetes (the therapeutic class of SGLT2 inhibitors, such as dapagliflozin, canagliflozin, empagliflozin). Phlorizin was not used in therapy due to several inadequacies: poor therapeutic selectivity, inhibiting both SGLT1 and SLGT2, poor stability being hydrolyzed by the intestinal phlorizin hydrolase, and low bioavailability. Interestingly, phloretin, the hydrolysis product of phlorizin, demonstrated GLUT-inhibitory properties [64]. So far, several natural compounds have been identified as potential SLGT1 or SLGT2 inhibitors in various structure-based simulated scanning studies: (−)-kurarinone and sophoraflavanone G isolated from *Sophora flavescens* roots [65], gneyulins A and B isolated from the bark of *Gnetum gnemonoides* [66], farnesal and farnesol acyclic terpenoids extracted from *Annona diversifolia* Safford leaves [67], phlorizin, (+)-pteryxin, (+)-ε-viniferin [68], formononetin, and (+)-pteryxin [69]. However, these compounds have been evaluated only as potential antidiabetic agents, and, therefore, supplementary cytotoxicity studies are needed to assess their role in tumor glycolysis and their anticancer potential.

### 5.3. Inhibitors of Hexokinase 2 (HK2)

HKs represent rate-controlling enzymes that kinetically regulate the glycolytic pathway. They catalyze the first step of glucose metabolism, being responsible for glucose phosphorylation to glucose-6-phosphate. The end product of the reaction is a precursor for both anabolic and catabolic processes: glycogenesis, hexosamine biosynthesis, glycolysis, and pentose phosphate pathway [70]. There are four HK isoforms, of which HK2 is highly expressed in malignant cells with enhanced aerobic glycolysis [70]. In fact, HK2 overexpression has been consistently reported in numerous types of cancer, including hepatocellular carcinoma, breast, colorectal, gastric, and lung cancer. Up to 80% of HK2 are localized in specific subdomains of interaction between mitochondria and endoplasmic reticulum called Mitochondria Associated Membranes (MAMs). Their localization was shown to contribute to the maintenance of intracellular and mitochondrial Ca^2+^ homeostasis, HK2 displacement promoting Ca^2+^-dependent death of cancer cells. In most cancer cells, mitochondrial HK2 promotes cell survival by interacting with the pore-like protein voltage-dependent anion channel (VDAC) to inhibit apoptosis [71]. 

Regulation of HK2 is mainly subjected to PI3K/Akt/mTOR pathway. HK2 is a transcriptional target of HIF-1α, stabilized under (pseudo)hypoxic conditions and associated with decreased oxidative phosphorylation and enhanced glucose consumption [72]. It is well known that HIF-1α expression is also under the control of the PI3K/Akt/mTOR pathway, confirming that its activation can lead to HK2 upregulation [70].

Of the natural compounds that demonstrated HK2 inhibitory properties, flavonoids take an important place. Xanthohumol, a prenylated flavonoid from *Humulus lupulus*, inhibited the proliferation of HCT116, HT-29, and SW620 colorectal adenocarcinoma cells in a time-dependent manner, at relatively low concentrations (5 µM). The proposed mechanism involved suppression of HK2 and glycolysis through direct inhibitory effects on epidermal growth factor receptor-protein kinase B (EGFR-Akt) signaling. Moreover, the immunoblotting data confirmed that the treatment markedly decreased HK2 also in a murine xerograph model [73]. In another in vitro/in vivo study, quercetin was shown to inhibit the proliferation of liver cancer cells by decreasing the HK2 protein level and suppressing the AKT/mTOR pathway. The effects of quercetin (12.5, 25, and 50 µM) were tested on glycolysis-addicted BEL-7402 and SMMC-7721 hepatocellular carcinoma cells, as well as on an SMMC-7721 xenograft model [74]. Similar results were obtained following exposure of non-small cell lung cancer cell lines (H460, HCC827, and H1650) to deguelin, a naturally occurring flavonoid from *Mundulea sericea*. Deguelin decreased HK2 expression by down-regulating Akt phosphorylation, confirming that the Akt signaling pathway is involved in the HK2 regulation. Additionally, results from the Western blot and immunohistochemical staining indicated that deguelin promoted H460 cells apoptosis in vivo [75]. According to another study, chrysin, a bioactive flavone derived from blue passionflower (*Passiflora caerulea*) (60 µM, 24 h), triggered cell apoptosis in hepatocellular carcinoma cells following HK2 reduction. Moreover, overexpression of HK2 attenuated the effect of chrysin on apoptosis and tumor glycolysis, pointing out that HK2 plays an important role in chrysin-mediated glycolysis suppression [76].

Using a more direct mechanism, oroxylin A, an O-methylated flavone isolated from *Scutellaria* root, was shown to inhibit glycolysis in MDA-MB-231 and MCF7 breast adenocarcinoma cells. The proposed mechanisms involved increased expression and mitochondrial translocation of sirtuin-3, leading to deacetylation of cyclophilin D and further detachment of HK2 from the mitochondria [77]. In a similar experimental design, dioscin (1, 2, 5 µM), a steroidal saponin, inhibited glycolysis and induced cell apoptosis in HT-29, HCT-116, and SW480 colorectal adenocarcinoma cells via promoting c-Myc ubiquitination and subsequent HK2 suppression [78].

In the evolution of hepatocellular carcinoma, activation of hepatic stellate cells (HSC) plays an essential role. Costunolide, a sesquiterpene lactone first isolated from costus (*Saussurea lappa* Clarke), was reported to reduce the viability and activation of HSCs by blocking aerobic glycolysis via HK2 inhibition [79]. Using RT-PCR and western blot, the lactone was shown to downregulate both mRNA and protein expression of HK2 in a concentration-dependent manner.

In addition to these natural compounds that have been shown to successfully block HK2 in various in vitro studies, other compounds are constantly being sought through computational techniques.

In a recent molecular docking study for potential HK2 inhibitors from *Andrographis paniculate* and *Centella asiatica*, the phytocompounds with binding energy lower than the one of glucose 6-phosphate, used as control, were further considered for post docking analysis, drug-likeness properties, and molecular dynamics simulations. Based on Ser-657, Thr-680, and Ser-897 interactions, 11 natural molecules showed good potential for HK2 binding. Of these, bayogenin, asiatic acid, and andrographolide were further confirmed as ligands of HK2 through molecular dynamics simulation [80]. Bao et al. [81] applied structure-based virtual ligand screening of a natural-compound database for HK2 inhibitors. Screening results predicted that (22E,24R)-6β-methoxyergosta-7,9(11),22-triene-3β,5α-diol, a steroid derived from *Ganoderma sinense*, has a high binding affinity for HK2. Further, the compound, together with the other 12 steroid analogs, was tested using in vitro microscale thermophoresis, enzyme inhibition, and other cell-based assays. The identified steroid exhibited a 4-fold selectivity against SW1990 human pancreatic cancer cells, suggesting a possible effect on HK2. However, more detailed anti-tumor action mechanisms are awaited.

### 5.4. Inhibitors of Phosphofructokinase 1 (PFK-1)

Another key regulatory enzyme in the glycolytic pathway is PFK-1. PFK-1 catalyzes the conversion of fructose-6-phosphate into fructose-1,6-bisphosphate along with the irreversible conversion of ATP to adenosine diphosphate (ADP) [82]. Three isoforms of PFK-1 have been described so far, liver isoform (PFKL), muscle isoform (PFKM), and platelet isoform (PFKP). All isoforms are present in the brain, while PFKP and PFKM are specific to platelets and adult muscles, and PFKL is liver and kidney-specific. PFK-1 is activated in the presence of a low ATP/adenosine monophosphate (AMP) ratio, and it is inhibited by increased acidic conditions to prevent excessive lactic acid production [83]. In the cytoplasm, PFK-1 molecules are activated by fructose-2,6-bisphosphate, AMP, and ADP and inhibited by citrate, phosphoenolpyruvate, and ATP. Upper regulation of PFK-1 activity occurs due to different oncogenes such as Ras or Src, allosteric activators, or by posttranslational modifications, which may include AKT/PI3K activation [84,85].

As PFK-1 sustains an increased glycolysis rate, an overexpression of PFK-1 was detected in several cancers such as breast cancer, ascites tumors, glioblastoma, B cell, and T cell leukemias, in which PFKP was identified as the predominant isoform [85]. Recently, stable knockdown of PFK-1 expression was shown to inhibit cell growth, induce apoptosis, decrease the invasive capability and metastasis of a nasopharyngeal carcinoma cell line, underlying the potential of targeting PFK-1 in different malignant tumors [86].

One of the first studies which investigated the effect of a natural compound on PFK-1 activity used resveratrol (1, 5, 15, 50, and 100 µM), a polyphenol with already demonstrated antitumor properties. In MCF7 breast cancer cells, resveratrol decreased glucose consumption and ATP content, effects which were directly correlated with PFK-1 inhibition. Moreover, resveratrol was shown to directly inhibit purified PFK-1 by promoting its dissociation from fully active tetramers into less active dimers. In the presence of PFK-1 negative regulators such as ATP and citrate, the effect was amplified, while positive PFK-1 modulators such as fructose-2,6-bisphosphate and ADP hindered the inhibitory activity of resveratrol [87].

Another flavonoid compound, bergapten, a derivative of psoralen found in bergamot essential oil, caused similar effects in MCF7 and ZR75-1 breast cancer cells. Doses of 20 and 50 μM decreased PFK-1 expression and lactate production rates, promoting glucose accumulation [88,89].

The most effective catechin in green tea, EGCG, was shown to inhibit cell growth and induce apoptosis by directly suppressing the PFK activity. The identified mechanism implied the transformation of the oligomeric structure of PFK into its inactive form. Moreover, when the hepatocellular carcinoma cell lines were also exposed to a PFK inhibitor, the effect was enhanced, while the cotreatment with an allosteric activator reversed the EGCG-induced cell death [90].

Similarly, sulforaphane, an isothiocyanate commonly found in broccoli and other cruciferous vegetables, was shown to induce apoptosis in hepatic cancer cells through inhibition of 6-phosphofructo-2-kinase/fructose-2,6-biphosphatase 4 (PFKFB4). The study provided evidence that sulforaphane triggered HIF-1 pathway inhibition, presumably mediated through MAPK. The study design is a valuable example of how proteomics can be employed for investigating the anticancer mechanisms of natural compounds. Following sulforaphane treatment (20, 40, and 60 μM), two specific proteomics techniques were performed, namely two-dimensional electrophoresis (2-DE)-based-proteomic analysis for total protein profiling and matrix-assisted laser desorption/ionization-time of flight mass spectrometry (MALDI-TOF MS) for the identification of differentially expressed proteins [91].

The same HIF-1 inhibitory effect was also proposed for worenine, an isoquinoline alkaloid isolated from *Coptis chinensis*, in a study designed to explore its pharmacological effects and mechanisms against colorectal cancer cells. Worenine (5, 10, and 20 µM) significantly decreased the protein and mRNA levels of several important glycolytic enzymes, including PFKL, by negatively regulating HIF-1α via Von Hippel–Lindau tumor suppressor protein (p-VHL) activation [92]. A similar decrease in HIF-1 and glycolysis enzymes HK2 and PFK-1 was triggered by the oleanolic acid (10, 20, and 30 μM), a triterpenoid widely found in plants of the Oleaceae family. However, in this case, the inhibition of HIF-1α-mediated glycolysis in gastric tumor cells required the disruption of yes-associated protein signaling [93].

Prosapogenin A, a saponin extracted from *Veratrum* sp., reduced the expression of the signal transducer and activator of transcription 3 oncogene (STAT3), GLUT1, HK, and PFKL mRNA levels at a concentration of only 5 µM. The effect of prosapogenin A was evaluated on HeLa cervical adenocarcinoma cells, Hep G2 hepatocellular carcinoma, and MCF7 breast adenocarcinoma cells, in comparison with 7701 and 293 non-cancer cells. In all tumoral cells, the saponin treatment inhibited cell growth and promoted apoptosis by modulating the expression of glycometabolism-related genes [94].

### 5.5. Inhibitors of Pyruvate Kinase (PKM)

Pyruvate kinase (PKM) plays a vital role in the final step of glycolysis, where it catalyzes the rate restrictive conversion of phosphoenolpyruvate to pyruvate with the production of one ATP molecule. Of the four PKM isoforms, PKM1 is found in high-energy demanding tissues, while PKM2 is expressed mainly in high proliferating cells like embryonic tissue and tumor cells. All isoforms exist in a stable tetrameric configuration, except PKM2, which may switch between a dimeric form with low PK activity, and a tetrameric form, with high PK activity, depending on the environmental conditions and the metabolic needs of the cells. Due to the PKM2 role in tumor tissue repair and regeneration, the enzyme has received increasing attention [95].

Apparently, PKM2 can translocate to the nucleus, where it regulates the transcription of numerous genes through HIF-1α, β-catenin, signal transducers, and activators of STAT3 and other transcription factors, triggering inflammatory response-signaling cascades and cell malignant transformation. Also, PKM2 was shown to promote histone H3 phosphorylation, favoring mitotic chromatin condensation and upregulating the transcription of cell-cycle-regulating genes such as c-Myc and Cyclin D1 [96]. The expression of PKM2 is upregulated in numerous cancer types, such as lung, breast, kidney, cervix, papillary thyroid, and others [97].

PKM2 expression is regulated by numerous factors, including heterogeneous ribonucleoproteins, peroxisome proliferator-activated receptor γ (PPARγ), epidermal growth factor receptor (EFGR), non-coding RNAs, and c-Myc protein. However, an important role in PMK2 regulation is played by HIF-1α activation through direct regulation of the c-Myc/hnRNP splicing axis or via PI3K/mTOR signaling pathway [97].

Several natural compounds have been identified as potent PMK2 inhibitors, suppressing aerobic glycolysis in cancer cells.

One of the best-known isoquinoline alkaloids, berberine, suppressed HCT-116 colon cancer cell and HeLa cervical cancer cell proliferation in a dose-dependent manner (IC_50_ value 63.6 μM and 157.4 μM, respectively). The presumed molecular mechanism was inhibition of PKM2 activity (no effect on PKM2 expression), as shown by immunoblot analysis and pyruvate kinase assay. Moreover, the study showed that the intermolecular forces between berberine and PKM2 were hydrogen bonding, as revealed by fluorescence spectroscopy [98].

Of the glycosyloxyflavone class, scutellarin has proved to have potent inhibitory effects against PKM2. Briefly, scutellarin directly targeted PKM2 and inhibited its cytosolic activity by decreasing glycolytic metabolism. On the other hand, scutellarin may also participate in regulating the cell cycle and apoptosis by activating MEK/ERK/PIN1 signaling pathway to promote the nuclear translocation of PKM2 [99]. In a more recent study, inhibition of PKM2 by scutellarin led to the resensitization of oxaliplatin-resistant colorectal cancer cells to oxaliplatin treatment. Mechanistically, scutellarin inhibited the PKM2 expression, reducing the glycolytic rate and the ATP production, which led, subsequently, to mitochondrial dysfunction [100].

Gliotoxin, a marine-derived fungal secondary metabolite, was found to directly bind PKM2 with high affinity in a dose-dependent manner, inhibiting glucose metabolism and triggering cell apoptosis. The inhibitory properties of gliotoxin were confirmed by surface plasmon resonance assay. Moreover, gliotoxin suppressed tyrosine kinase activity of PKM2, leading to a dramatic reduction in STAT3 phosphorylation, validating, in this way, the link between PKM2 and STAT3. Further, the gliotoxin effect on proliferation of several cell lines representative of colorectal and breast cancers, prostate and lung adenocarcinoma, leukemia, and glioblastoma was tested. The most sensitive cells were U87 glioblastoma cells (IC_50_ value of 0.54 μM, MTT assay, 72 h). This cell line was further used in cellular thermal shift assay (CETSA), where the direct binding between gliotoxin and PKM2 was confirmed [101]. Another fungal secondary metabolite, pseurotin A, exhibited a similar mechanism in glioma cells, downregulating the expression level of PKM2 [102].

Shikonin, a naturally occurring naphthoquinone extracted from *Lithospermum erythrorhizon*, significantly inhibited the glycolytic flux in several cancer cells (MCF7, MCF7/Adr, MCF7/Bcl-2, MCF7/Bcl-xL, and A549). First, glucose consumption and lactate production assays were performed. Next, solid-phase bonded shikonin was used to extract cellular proteins that were further analyzed by mass spectroscopy. Shikonin and its enantiomer, alkannin, were found as potent and specific inhibitors to PKM2 without affecting the activity of LDH [103].

Chai et al. identified *Carpesium abrotanoides* (L.) root as a source of natural compounds targeting glucose metabolism. The group studied the effect of an ethanolic root extract on MCF7 and MDA-MB-231 breast adenocarcinoma cell proliferation, apoptosis, and migration. Results indicated a dose-dependent anti-proliferative effect on both cell lines, the mechanism involving downregulation of PKM2 expression. The root extract altered PKM2 cellular translocation by blocking the PKM2/HIF-1α axis. The observed effects were not attributed to a specific compound but rather to a group of active compounds, which were identified using MS analysis [104]. Similarly, an olive leaf extract enriched in oleuropein determined a 50% reduction of mRNA and protein levels corresponding to GLUT1, PKM2, and MCT4, triggering cell growth inhibition in tumor cells representative of melanoma [105].

### 5.6. Inhibitors of Lactate Dehydrogenase (LDH)

LDH belongs to the oxidoreductase class, and it is responsible for the reversible conversion of pyruvate to lactate and the concomitant oxidation of NADH to NAD+ and vice versa. LDH has a tetrameric structure composed of M and H subunits that define its five isoforms (LDH-1, LDH-2, LDH-3, LDH-4, and LDH-5). The isoforms consisting of M-subunits, such as LDH-5 convert pyruvate to lactate, while isomers with a substantial proportion of H-subunits catalyze the reverse reaction. Functional regulation of LDH at the transcriptional level is influenced by methylation and transcription factors such as c-Myc and HIF-1α, mostly activated by PI3K/Akt/mTOR pathway. An LDH-5 elevated expression was observed in several cancers: non-small cell lung cancer, gastric cancer, and melanoma. Reduction of LDH-5 in Warburg-shifted cancer cells was shown to induce apoptosis, as NAD+ generation was disrupted, thus validating LDH-5 as a promising therapeutic target [106].

Gossypol, a natural phenol isolated from cotton seeds, was shown to possess spermicidal activity due to its ability to inhibit spermatozoal lactate dehydrogenase-X (LDH-X) [107]. Also, wogonin, already recognized as a potent anticancer flavonoid, was shown to affect proliferation and the energy metabolism in gastric (SGC-7901) and lung (A549) cancer cells by inhibiting the LDH activity [108].

In a recent study, a screening of LDH inhibitors from bioactive compounds was carried out using integrated electrophoresis-mediated microanalysis, transverse diffusion of laminar flow profiles, and rapid pressure direction switching. The screening results confirmed gossypol as a potent LDH inhibitor (IC_50_ = 9.269 µM) and identified quercetin, luteolin, and ursolic acid as potential inhibitory agents. Moreover, these natural compounds bind well to the residues of the active pocket of LDH, as confirmed by molecular docking studies [109]. In fact, the inhibitory capacity of a luteolin conjugate was previously demonstrated using LDH assay and molecular modeling studies. More precisely, luteolin 7-O-β-d-glucoside, found in *Phlomis kurdica*, nonspecifically inhibited both LDH-1 and LDH-5 [110].

Similarly, in a virtual screening study designed to identify novel LDH inhibitors, galloflavin, obtained by gallic acid oxidation, showed total inhibition of both LDH-A and LDH-B. The effect was confirmed following exposure of PLC/PRF/5 hepatocellular carcinoma cells to galloflavin. The proposed mechanism involved a direct binding of the free enzyme without competing with the substrate and without interfering with cell respiration [111].

The same strategy was implemented in the study of *Polygala flavescens*, where 3,6′-di-O-sinapoylsucrose and reiniose F were identified as potent LDH-5 inhibitors, with reported IC_50_ values of 90.4 μM and 190.7 μM, respectively [112]. Wang et al. [113] validated epigallocatechin’s effects on LDH-A activity and expression by in vitro enzymatic activity assay and western blotting. The authors implemented a bioactivity-guided fractionation strategy on *Spatholobus suberectus*, obtaining a subfraction enriched in catechins. From this subfraction, epigallocatechin exhibited the strongest LDH-A inhibition and apoptosis induction effect. Gallocatechin, catechin, and epicatechin showed synergistic LDH-A suppression effects when associated with epigallocatechin. The ester of epigallocatechin and gallic acid, epicatechin-3-O-gallate, was also identified as a potent LDH inhibitor in a very interesting approach implemented by Cheng et al. [114]. The group developed a screening method using LDH functionalized magnetic nanoparticles and used them for “fishing” for LDH inhibitors from *Rhubarb* and *Polygonum cuspidatum*. The study resulted in nine LDH inhibitors tentatively identified by MS: (−)-epicatechin-3-O-gallate, ω-hydroxyemodin, emodin-1-O-gluciside, lunatin, chrysophanic acid, rhein-O-(acetyl)-glucoside, aloe-emodin, rhein, and emodin.

Recently, in silico ligand binding virtual screening was applied by Martin et al. [25] to find potential molecular targets for phytocannabinoids. The screening predicted an interaction of phytocannabinoids with the LDH enzymes, an interaction which was confirmed in vitro, with cannabichromene (CBC) and Δ^9^-tetrahydrocannabinolic acid (Δ^9^-THCA) noncompetitively inhibiting the LDH-A enzyme function by more than 50%. A similar noncompetitive inhibition mechanism was described also for resveratrol [115]. 

**Figure 2:**
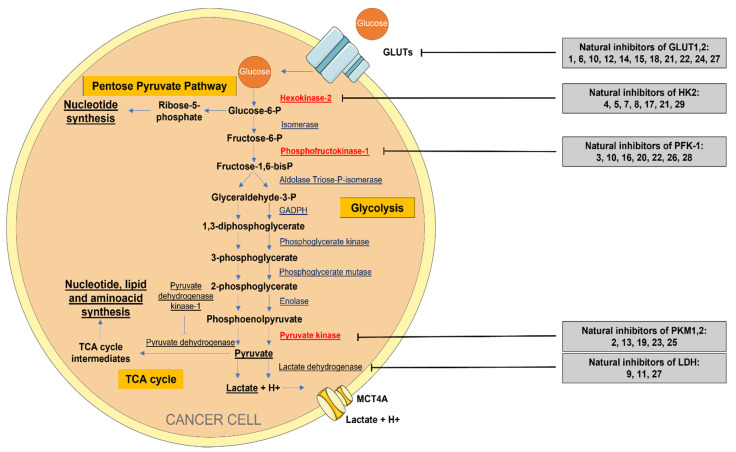
Simplified scheme of the energetic metabolism in cancer cells. Aerobic Glycolysis, Pentose Pyruvate Pathway, and TCA cycle. Natural compounds which inhibit the glycolytic pathway according to in vitro studies (the ligand-binding screening studies without in vitro testing were excluded): **1.** Apigenin, **2.** Berberine, **3.** Bergapten, **4.** Chrysin, **5.** Costunolide, **6.** Daidzein, **7.** Deguelin, **8.** Dioscin, **9.** Epigallocatechin, **10.** Epigallocatechin gallate, **11.** Galloflavin, **12.** Genistein, **13.** Gliotoxin, **14.** Glucopiericidin A, **15.** Kaempferol, **16.** Oleanolic acid, **17.** Oroxylin A, **18.** Phloretin, **19.** Pseurotin A, **20.** Prosapogenin A, **21.** Quercetin, **22.** Resveratrol, **23.** Scutellarin, **24.** Silybin, **25.** Shikonin, **26.** Sulforaphane, **27.** Wogonin, **28.** Worenine, **29.** Xanthohumol. The red-colored enzymes represent key regulators of the glycolytic pathway.

All the above examples prove that phytochemicals can successfully interfere in regulating multiple steps of the glycolytic flux. Regardless of the targeted transporter/enzyme, the overall effect of these natural compounds was a clear inhibition of tumor glycolysis-associated proliferation, exerting promising antitumoral properties. An overview of the natural regulators of glycolysis, investigated through in vitro techniques, is presented in Figure 2 and summarized in Table 2.

## 6. Tumor Hypoxia and Their Natural Regulators

### 6.1. Hypoxia-Inducible Factor 1-alpha (HIF-1α) Regulating Signaling Pathway

The cellular reprogramming of glucose metabolism involves transcriptional upregulation of glucose membrane transporters and glycolytic enzymes, as presented above. The increased glucose uptake and flux through the pathway allow cells to maintain a sufficient ATP production, avoiding mitochondrial OXPHOS. This reprogramming of glucose metabolism is achieved by the HIF-1 transcription factor, which plays a critical role in controlling the response of tumors to hypoxia.

HIF consists of a heterodimer composed of alpha (α) and beta (β) subunits with a basic helix-loop-helix structure [116]. HIF-1 is sensitive to the environmental oxygen level through its alpha subunit: in the presence of oxygen, HIF-1α is hydroxylated by prolyl hydroxylase domain protein 2 (PHD2), bound by the von Hippel-Lindau tumor suppressor protein (VHL) and subjected to proteasomal degradation [117]. Inactivation of HIF-1 in the presence of oxygen can also take place in a non-VHL-dependent manner: factor inhibiting HIF-1 (FIH-1) hydroxylases the HIF-1α C-TAD domain and hinders the interaction between C-TAD and HIF-1α target genes. In the absence of this interaction, HIF-1 is not able to activate the transcription of its target genes [118].

However, HIF-1α regulation takes place independent of the oxygen levels [119]. Upstream regulators of HIF-1α include diverse growth factors that can control its transcription or translation. Most of the effects are mediated by upstream activation of PI3K, which triggers a signaling cascade composed of serine/threonine kinases AKT and mammalian target of rapamycin (mTOR) [120]. On the other side, RAS, followed by MAP/ERK kinase (MEK), activates the extracellular-signal-regulated kinase (ERK). Both ERK and mTOR phosphorylate the eukaryotic translation initiation factor 4E (eIF-4E) binding protein (4E-BP1), impeding the interaction between the two molecules, which causes enhanced translation of HIF-1α [118].

**Figure 3:**
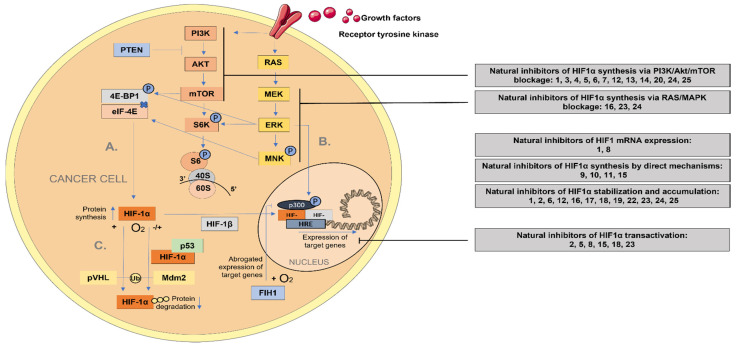
HIF-1α signaling pathways and their natural regulators. **A.** HIF-1α translation via PI3K/AKT/mTOR and RAS/MEK/ERK pathways **B.** ERK regulation of the HIF transcription regulatory complex. **C.** Degradation of HIF-1α via pVHL mediated ubiquitination (in the presence of oxygen) and via p53/Mdm2 mediated ubiquitination (independent of oxygen). In normoxia, FIH-1 hydroxylates HIF-1α and impedes its transactivation. Natural compounds which inhibit the HIF-1 pathway: **1.** Apigenin, **2.** Biochanin A, **3.** Berberine, **4.** Baicalein, **5.** Curcumin, **6.** Chrysin, **7.** Chlorogenic acid, **8.** Cryptotanshinone, **9.** Deguelin, **10.** Dictamnine, **11.** Epigallocatechin-3-gallate (EGCG), **12.** EGCG and green tea extract (GTE), **13.** Galangin, **14.** Gambogic acid, **15.** Genistein, **16.** Kaempferol, **17.** Licochalcone A, **18.** Luteolin, **19.** Magnolol, **20.** Myricetin, **21.** Oridonin, **22.** Oroxylin A, **23.** Quercetin, **24.** Resveratrol, and **25.** Wogonin.

The regulatory activity of HIF-1α is initiated in the nucleus through the formation of a regulatory complex (composed of HIF-1β, p300, and others). This complex binds to the hypoxia response elements (HRE) and initiates target gene expression (see Figure 3).

An overview of meta-analyses studies showed that HIF-1α expression was significantly associated with lymph node metastasis and the survival rate in a great number of malignancies, including osteosarcoma, lung, breast, esophageal, gastric, colorectal, pancreatic, and ovarian cancer. A high HIF-1α expression was correlated with lymph node metastasis but not with survival, in glioma, nasopharyngeal cancer, and renal cancer. Inversely, expression of HIF-1α associated only with survival was seen in oropharyngeal cancer, hepatocellular carcinoma, and cervical cancer. A more limited correlation with the same parameters was observed in the case of HIF-2α expression [121].

### 6.2. Natural Inhibitors of Phosphoinositide 3-Kinase/Protein Kinase B/Mammalian Target of Rapamycin (PI3K/Akt/mTOR)

The PI3K/Akt/mTOR pathway is of great importance in tumor progression, as it regulates diverse downstream processes such as transcription, translation, metabolism, proliferation, cell growth, angiogenesis, and metastasis [122]. The central mediator of the pathway is Akt, which was found to regulate multiple control points in glycolysis. The direct post-translational regulation of glycolysis can be accomplished by promoting glucose uptake through glucose transporters (mainly GLUT1 and GLUT4) or through the phosphorylation and activation of specific glycolytic enzymes (HK2, PFK-1).

As the PI3K pathway is the most activated route in the case of human cancers, finding specific blocking agents is highly desirable. In this light, multiple natural compounds have been identified as potent inhibitors of the PI3K/Akt/mTOR pathway, interfering in one or more key points.

Apigenin, a dietary flavonoid, was found to induce apoptosis and autophagy in Hep G2 hepatocellular carcinoma cells by decreasing the levels of phosphorylated PI3K, Akt, and mTOR, while pretreatment with a specific PI3K inhibitor reinforced Hep G2 cells sensitivity to apigenin [9]. The same apoptotic effect was observed after A375SM human melanoma cells were exposed to 50 and 100 μM apigenin for 24 h. The treatment increased extracellular signal-regulated kinase (ERK) and c-Jun N-terminal kinase (JNK) and decreased p38 and Akt protein expression levels, suggesting that Akt and MAPK pathways were affected [123].

Biochanin A, one of the predominant isoflavones in *Trifolium pratense*, inhibited U251 glioblastoma growth via restricting glycolysis and mitochondrial OXPHOS. Phosphorylation of AKT and mTOR, along with decreased expression of HIF-1α, GLUT1, HK2, and LDH-A, indicated that Akt/mTOR/HIF-1α inhibition is responsible for the decreased glycolytic capacity [124].

An isoquinoline alkaloid, berberine, inhibited the overactive glucose metabolism of HCT116 and KM12C colorectal cancer cells by suppressing mTOR-dependent HIF-1α protein synthesis. Also, berberine inhibited the transcription of glucose metabolism-related genes, GLUT1, LDH, and HK2, in a concentration-dependent manner [125]. Similar results were observed on U251 and U87 glioma cells, where berberine attenuated glycolysis-dependent energy production and induced mitochondrial dysfunction by targeting the AMPK/mTOR/ULK1-pathway [126].

Curcumin triggered down-regulation on PKM2 via mTOR-HIF-1α inhibition in H1299 non–small cell lung cancer cells, MCF7 breast adenocarcinoma cells, HeLa cervical adenocarcinoma cells, and PC-3 prostate adenocarcinoma cells, reversing the Warburg effect [127]. Moreover, Wang et al. found that curcumin inhibited aerobic glycolysis in HCT116 and HT-29 colorectal carcinoma cell lines and induced mitochondrial-mediated apoptosis through HK2 in an Akt-dependent manner [128].

Licochalcone A, a chalcone derived from licorice, triggered glycolysis inhibition and decreased HK2 expression in MKN-45 and SGC-7901 gastric cancer cells. The mechanisms were mainly attributed to the blockade of the Akt signaling pathway, while hyperactivation of Akt attenuated licochalcone A-mediated suppression [129]. Similarly, in MCF7 breast adenocarcinoma cells, licochalcone A inhibited PI3K/Akt/mTOR activation, promoting autophagy and apoptosis [130].

Resveratrol, a widely studied polyphenol, was shown to inhibit the proliferation of A2780 and SK-OV-3 ovarian cancer cells by blocking glycolysis and decreasing the expression and activation of AMPK downstream kinase mTOR [131]. Using a similar mechanism, resveratrol impaired glucose metabolism also in non-small cell lung cancer cells, decreasing HK2 expression via targeting the Akt signaling pathway [132].

Similarly, xanthohumol induced HK2 and glycolysis inhibition in colorectal cancer cells, both being partly dependent on the suppression of Akt activation [73]. Also, glioblastoma cells exposed to xanthohumol resulted in inhibition of HK2 expression and impaired tumor glycolysis. The identified mechanism involved down-regulation of the PI3K/Akt-GSK3β-FBW7 signaling axis, which promoted the destabilization of c-Myc, required for a xanthohumol-induced decrease in HK2 [133].

### 6.3. Natural Inhibitors of Ras/Raf/MAPK Signaling Pathway

Apart from PI3K/Akt/mTOR pathway, HIF-1α is constitutively transcribed and synthesized through another signaling pathway activated by growth factors and independent of the oxygen level, the Ras/Raf/MAPK signaling pathway (Figure 3).

More precisely, the ERK1/2-dependent phosphorylation was shown to activate HIF-1α and promote the Warburg effect by means of a pyruvate kinase M2-dependent positive feedback loop on its own expression as well as the expression of GLUT1 and LDHA [134]. A detailed review of the role of the ERK pathway in metabolic reprogramming, including the Warburg effect, was performed by Papa et al. [135].

Several natural compounds have been identified as promising therapeutic agents against Ras/Raf/MAPK signaling-driven cancers, impairing HIF-1α formation.

Quercetin was shown to enhance hypoxia-mediated apoptosis via direct inhibition of AMPK activity in HCT 116 colorectal cancer cells. The proapoptotic effects of quercetin were superior in hypoxic conditions than in normoxic settings. Moreover, quercetin significantly inhibited HIF-1 transcriptional activity without altering the mRNA and protein levels of HIF-1α and sensitized HCT116 cancer cells to cisplatin and etoposide under hypoxic conditions [136]. Along with quercetin, luteolin and kaempferol were shown to inhibit HIF-1α phosphorylation and suppress its transcriptional activity by interfering with the MAPK pathway in HeLa cervical adenocarcinoma cells. The same study concluded that MAPK-pathway inhibition by the three tested flavonoids predominantly affects the nucleocytoplasmic transport of HIF-1α rather than its ability to interact with DNA, ARNt, or the coactivator CBP/p300 [137]. Kaempferol also proved to be a potent inhibitor of Huh-7 hepatocellular cancer cells under hypoxic conditions. The mechanisms involved p44/42 MAPK pathway inhibition and inactivation of HIF-1α by mislocalization, leading to decreased HIF-1α activity. Again, the flavonoid suppressed hepatocarcinoma cell survival more efficiently under hypoxia than under normoxic conditions [138].

The soy isoflavones, genistein and daidzein, were also shown to affect the tyrosine kinases signaling pathways, but their precise mechanisms are strongly correlated with the exposure dose and the estrogen receptor status [139].

An overview of the natural regulators of HIF-1α synthesis (by targeting upstream pathways, such as PI3K/Akt/mTOR pathway or Ras/Raf/MAPK signaling pathway), HIF-1α mRNA expression, stabilization, accumulation, and transcriptional activity are summarized in Table 3. In addition, an alphabetical listing of all these natural compounds, along with others, such as baicalein [140], myricetin [141] or oridonin [142] and their precise mechanism of action, can be found in the Supplementary material, Table S1. In Figure 3, we depicted the mechanisms of action of HIF-1α natural inhibitors, blocking different steps of the HIF-1α signaling pathway.

## 7. Conclusions

Substantial experimental evidence upholds the potential of plant-derived compounds to interfere in tumor glycolysis or HIF-1-mediated signaling pathways. Herein, we performed a structural classification of the phytochemicals involved in regulating these pathways, and we identified specific inhibitors for each biochemical checkpoint where the carcinogenic process can be interrupted: the glucose membrane transporters (GLUT1 and 2), the rate-limiting enzymes HK2, PFK-1, PKM2, along with other important enzymes such as LDH. Moreover, we review how phytochemicals can interfere in HIF-1 synthesis, stabilization, accumulation, and transactivation, emphasizing PI3K/Akt/mTOR and Ras/Raf/MAPK pathways as essential signaling cascades in HIF-1 activation.

In future perspectives, a bold change of optics concerning how phytochemicals are investigated in cancer-related models can be already observed. Due to the novel in silico approaches, the search for the most suitable anti-cancer agents starts from the structural configuration of the targeted molecule (transporter/enzyme) and uses bioinformatical tools such as molecular docking studies, drug-likeness property experiments, pharmacophore modeling, or molecular dynamics simulation studies in order to predict, as accurate as possible, new potential agents. As an example, for LDH, molecular docking studies have suggested gossypol, quercetin, luteolin, and ursolic acid [109] luteolin 7-O-β-d-glucoside [110], 3,6′-di-O-sinapoylsucrose, reiniose F [112], cannabichromene and Δ9-tetrahydrocannabinolic acid [25] just to mention a few natural potent ligands. However, the potential candidates obtained from in silico screenings must be confirmed in further in vitro/in vivo studies. One of the most accurate approaches that allow robust quantification of fluxes along the altered biochemical pathways is cell culture-based metabolomics, with its two main analytical platforms, nuclear magnetic resonance spectroscopy and high-resolution mass spectrometry.

The emerging power of omics technologies, along with computational simulation tools, offer unprecedented opportunities for understanding phytochemical mechanisms of action toward plant-derived drug discovery.

## Figures and Tables

**Table 1:** Non-targeted metabolomics studies evaluating the metabolic alterations induced by phytochemicals in various in vitro models.

Natural Compound/Extract	In Vitro Model	Study Design	Analytical Methods	Effect	Altered Pathways	Ref
Rosmary extract containing 256 µg/mL carnosic acid and 37.1 µg/mL carnosol Standard carnosic acid	Colorectal adenocarcinoma cells (HT-29)	9.9 µg/mL standard carnosic acid for 48 h	CE-TOF MS and HILIC/UHPLC-TOF MS	↑ GSH ↓ GSSG ↑ GSH/GSSG ratio ↓ N-acetylputrescine	glutathione metabolism, polyamine metabolism	[29]
Rosemary extract containing 226.39 µg/mg carnosol and 51.55 µg/mg carnosic acid	Colorectal adenocarcinoma cells (HT-29)	10 µM rosemary phenols	CE-ESI-TOF MS HILIC/UPLC-ESI-TOF MS	↓ GSH/GSSG ratio	glutathione metabolism, polyamine metabolism, urea cycle and metabolism of amino groups	[30]
Five rosemary phenolic extracts	Chronic myelogenous leukemia cells (K-562) and daunomycin-resistant chronic myelogenous leukemia cells (K-562/R)	5, 10 µM	CE-TOF MS and UPLC-TOF MS	↑ methionine ↑ leucine ↑ glutamine ↑ tyrosine ↓ lysine	aminoacyl-tRNA biosynthesis, glutathione metabolism, arginine, proline metabolism, nitrogen metabolism, urea cycle	[31]
Ionic liquid-Graviola fruit pulp extract	Colorectal adenocarcinoma cells (HT-29)	10.56 μg/mL for 48 h	GC-TOF MS	↑ thiocyanic acid ↓ lactic acid ↓ L-alanine ↓ tricosadiynoic acid	amino acid metabolism, aerobic glycolysis, urea cycle, ketone bodies metabolism	[32]
Soy seed extract (SSE), standard genistein (Gen), standard daidzein (Dai)	Breast adenocarcinoma cells (MCF7, MDA-MB-231)	For MCF7 cells: 23 μM Gen, 52 μM Dai, 166 μg/mL SSE; For MDA-MB-231 cells: 11 μM Gen, 36 μM Dai, 26 μg/mL SSE	^1^H-NMR GC-MS	↓ glucose uptake ↓ glutamine uptake	glucose transport, glycolysis, protein biosynthesis	[33]
Extra virgin olive oil extract	Colorectal adenocarcinoma cells (HT-29, SW480)	0.01–0.1%	nanoLC-ESI-TOF MS		cell cycle, metabolism of polyphenols	[34]
Resveratrol	Hepatocellular carcinoma cells (Hep G2)	40 μM	^1^H-NMR	↓ use of glucose and amino acids ↓ lactate release ↑ succinate use	glycolytic activity, energy production	[35]
	Breast adenocarcinoma cells (MCF7, MDA-MB-231)	5–100 mM for 72 h	LC-MS (Targeted approach)	↑ amino acids levels, ↑ serotonin, kynurenine, and putrescine synthesis	biogenic amine metabolism, arachidonic acid pathway	[36]
Curcumin	Breast adenocarcinoma cells (MCF7, MDA-MB-231)	0.5, 2.5, 10, 25, and 50 mg/L for 24 h for dose-dependent effect analysis; 10 mg/L for 24, 48, 72, and 96 h for the analysis of the time-dependent response	^1^H-NMR	-biphasic effect (≤28 μM, ↑ total GSH; ≥70 μM ↓ total GSH) -at high doses: accumulation of polyunsaturated and total free fatty acids, decrease in glycerophospho-ethanolamine and choline	glutathione metabolism, lipid metabolism	[37]

^1^H-NMR—Proton nuclear magnetic resonance spectroscopy, CE-ESI-TOF MS—Capillary electrophoresis–electrospray ionization–time of flight–mass spectrometry, CE-TOF MS—Capillary electrophoresis–time of flight–mass spectrometry, GC-TOF MS—gas chromatography-time of flight–mass spectrometry, GSH—Glutathione, GSSG—Glutathione disulfide, HILIC/UHPLC-TOF MS—HILIC/ultra-high pressure liquid chromatography-time of flight–mass spectrometry, HILIC/UPLC-ESI-TOF MS—HILIC/ultra-pressure liquid chromatography-electrospray ionization–time of flight–mass spectrometry, nanoLC-ESI-TOF MS—nano liquid chromatography–electrospray ionization–time of flight–mass spectrometry, LC-MS—liquid chromatography-mass spectrometry, UPLC-TOF MS—ultra pressure liquid chromatography electrospray ionization–time of flight–mass spectrometry. Up-arrow (↑) and down-arrow (↓) indicate an increase or a decrease, respectively, of the metabolite level following the phytochemical treatment.

**Table 2:** Natural compounds were identified to negatively regulate the glycolytic pathway in various cell culture models, along with the main information about the applied study design.

Natural Compound	In Vitro Models	Study Design	Ref
Inhibitors of GLUT transporters			
**Genistein** **Phloretin** **Apigenin** **Daidzein**	Prostate carcinoma cells (LNCaP) Normal prostatic epithelial cells immortalized with simian virus 40 (PNT1A)	Cell proliferation assay (Hoechst assay—48 h): 0 μM–100 μM for genistein and phloretin, 0 μM–50 μM for apigenin, 0 μM–140 μM for daidzein Glucose uptake (20 min, 1 h, 24 h) at IC_50_ Western blot for GLUT1,4 protein levels (6, 24, 48 h) at IC_50_ Immunocytochemistry for GLUT1,4 subcellular location (24, 48 h) at IC_50_ Molecular docking study (Target XylE 0 *E. coli* homolog of GLUT1-4)	[3]
**Silybin**	Colorectal adenocarcinoma cells (LoVo) Doxorubicin-resistant colorectal adenocarcinoma cells (LoVo-DOX)	Cell viability assay (MTT assay, 24, 48, 72 h) at 5, 10, 50 μM Western blot for GLUT1 protein levels (48 h) at 10, 50 μM	[50]
**Wogonin**	Colorectal carcinoma cells with different p53 expressions (HCT116, HT-29), normal colon epithelial cells (NCM460); Hepatocellular carcinoma cells with different p53 expressions (Hep G2, SMMC-7721), normal hepatic cells (L02); Ovarian cancer cells with different p53 expressions (A2780, SK-OV-3), normal ovarian cells (IOSE-80)	Cell viability assay (MTT assay, 24 h) Measurement of intracellular ROS Measurement of glucose uptake level and lactate generation ATP assessment Western blot for the protein expression and real-time PCR for the mRNA level of p53, TIGAR, HK2, LDH-A, PDK, GLUT1, and PGM In vivo tumor growth	[52]
**Resveratrol**	Acute promyelocytic leukemia cells (HL-60) and histiocytic lymphoma cells (U-937)	Glucose uptake at 0.1–100 μM resveratrol	[115]
**Glucopiericidin A**	Epidermoid carcinoma (A-431)	CE-MS metabolomics, cells grown in serum-reduced media for 18 h and treated for 30 min CE-MS [^13^C]-glucose labeling study Glucose uptake	[60]
**Bitter melon juice (MBJ, derived from *Momordica charantia* fruits)**	Pancreatic cancer cells (PANC-1, BxPC3)	Cell growth and viability assay (Trypan blue exclusion assay, 72, 96, 120, 144 h) NMR metabolomics analysis with 2% BMJ Immunofluorescence analysis of glucose and lactate transporter expression status after treatment with 2% BMJ	[61]
Inhibitors of hexokinase 2			
**Xanthohumol**	Colorectal carcinoma cells (FHC, CCD841 CoN, HT-29, SW480, LoVo, HCT116, and SW620)	Cell viability assay (MTS assay, 24, 48, 72 h) at 2, 4, 8 μM Glucose uptake and lactate production measurement Immunoblotting for HK2 expression In vivo tumor growth	[73]
**Quercetin**	Hepatocellular carcinoma cells (SMMC-7721, BEL-7402) Normal hepatic cells (LO2)	Cell proliferation assay (MTS assay, 24 h) at 12.5, 25, 50 µM Glucose uptake and lactate production assays Western blot assay for HK2 protein expression Western blot for the expression of p-Akt/Akt, p-mTOR/mTOR In vivo tumor growth and IHC staining	[74]
**Deguelin**	Non-small cell lung cancer cells (H460, H1650, H1299, H520, HCC827, H1975, and H358)	Cell viability assay (MTS assay, 24, 48, 72 h) at 1, 2, 5 μM Western blot assay for HK2 protein expression Glucose uptake and lactate production measurement In vivo tumor growth	[75]
**Chrysin**	Hepatocellular carcinoma cells (Hep G2, Hep3B, Huh-7, HCC-LM3, BEL-7402, and SMMC-7721) Normal hepatic cells (LO2)	Cell viability assay (Cell Titer-Glo assay, 0, 24, 48, 72 h) at 15, 30, 60 μM Western blot assay for HK2 protein expression Apoptosis assays Glucose uptake and lactate production measurement at 15, 30, and 60 μM In vivo tumor growth assay and IHC staining	[76]
**Orxoylin A**	Breast adenocarcinoma cells (MDA-MB-231, MCF7)	Cell viability assay (MTT assay, 48 h) at 0–250 μM Glucose uptake and lactate production measurement Western blot assays for PFKFB1/4, PFKFB2, PKM2, LDH and GLUT1 (at 150 μM) Western blot analysis for HK2 expression (at 100, 150, and 200 μM)	[77]
**Dioscin**	Colorectal carcinoma cells (HCT116, HT-29, DLD1, and SW620)	Cell viability assay (Cell Titer-Glo assay, 24, 48, 72 h) at 0, 1, 2, 5 µM Tumor glycolysis measurement Western blot analysis for HK2 protein expression Apoptosis assays In vivo tumor growth and IHC staining	[78]
**Costunolide**	Primary hepatic stellate cells	Cell viability assay (MTT assay, 24 h) at 10, 20, and 30 μM Glucose uptake and intracellular lactate Measurement of hexokinase activity Western blot assay for HK2 protein expression	[79]
**(22E,24R)-6β-methoxyergosta-7,9(11),22-triene- 3β,5α-diol**	Pancreatic ductal adenocarcinoma cells (SW1990) African green monkey kidney cells (Vero cells)	In vitro HK2 enzyme inhibition assay Molecular docking to human HK2 Microscale thermophoresis assay for the binding affinity of *Ganoderma sinense* potential ligands to target Cytotoxicity assay (Cell Counting Kit -8-48 h) to 0–100 μM	[81]
Inhibitors of phosphofructokinase 1 (PFK-1)			
**Resveratrol**	Breast adenocarcinoma cells (MCF7)	Cell viability assay (MTT assay, 24 h) at 0, 1, 5, 15, 50, and 100 µM Glucose consumption and lactate production assays LDH leakage, ATP content, and PFK activity assay Radioassay for PFK activity and PFK intrinsic fluorescence measurement	[87]
**Bergapten**	Breast cancer cells (MCF7, ZR75-1)	Western blot for PFK-1 expression (6 and 16 h, at 20 and 50 µM) Glucose and LDH assays Triglyceride and lipase activity assay G6PDH activity and ATP assay Isocitrate dehydrogenase and malic enzyme activity assay	[88]
**Epigallocatechin-3-gallate**	Hepatocellular carcinoma cells (HCC-LM3, Huh-7, Hep G2, Hep3B, SMMC-7721) Normal hepatic cells (LO2)	Glucose uptake, lactate production (25, 50, 100 μM) RT-PCR analysis for the expression of glycolysis-associated genes Western blot assay for PKF protein expression PFK activity (25, 50, 100, and 200 μM) Cell proliferation assay (CCK assay -8; 24 h; 25, 50, 100, 200 and 400 μM) Animal experiments	[90]
**Sulforaphane**	Hepatocellular carcinoma cells (Huh-7, SNU-449, and NCTC cells)	Cell proliferation assay (Real-time cell electronic sensing) Apoptosis assays, Cell cycle analysis Protein analysis (MS-based protein analysis and RT-PCR validation) PFK expression validation through immunoblot and RNAi technique	[91]
**Worenin**	Colorectal carcinoma cells (HCT-116, SW-620) Normal colon cells FHC (CRL-1831)	Cell viability assay (MTT assay, 24 h at 0, 1, 5, 10, 20, 40 or 80 μM) Colony formation test and cell cycle analysis Glucose consumption and lactate production assays Western blot for GLUT3, HK2, PKM2, and LDHA protein expressions RT-PCR analysis for GLUT3, HK2, PKM2, and LDHA mRNA expressions Western blot and immunofluorescence staining for nuclear HIF-1α expression	[92]
**Oleanolic acid**	Gastric cancer cells (MKN-45, SGC-7901) Normal gastric mucosal epithelium cells (GES-1)	Cell viability assay (MTT assay) and proliferation (BrdU incorporation) assays (24 h, at 0, 10, 20, 30 µM for the cancerous cells, and up to 80 µM for the normal cells) Glucose consumption and lactate production assays Western blot assay for HK2, PFK-1, HIF-1α, and YAP protein expression RT-PCR assay for HIF-1α, HK2, and PFK-1 mRNA expression	[93]
**Prosapogenin A**	Cervical adenocarcinoma cells (HeLa) Hepatocellular carcinoma cells (Hep G2) Breast adenocarcinoma cells (MCF7) Non-cancer cells (7701, 293)	Cell viability assay (MTT assay, 24, 48, and 72 h) at 10 μM Cell apoptosis and cell cycle analysis Western blot assay to detect the STAT3 mRNA protein level RT-PCR to detect the STAT3, GLUT1, HK, and PFKL mRNA level (5 µM, 48 h)	[94]
Inhibitors of pyruvate kinase (PKM)			
**Berberine**	Colorectal cancer cells (HCT-116) Cervical adenocarcinoma cells (HeLa)	Cell viability assay (MTT assay, 24 h) at 50–300 μM Western blot assay for PKM protein expression Pyruvate kinase assay (100–300 μM)	[98]
**Scutellarin**	Cervical adenocarcinoma cells (HeLa)	Biotinylated scutellareins as probes for target identification in HeLa lysate	[99]
	Colorectal adenocarcinoma cells (SW480, HT-29) Oxaliplatin-resistant colorectal cancer cells (OR-SW480, OR-HT-29)	Cell viability assay (MTT assay, 24, 48 h) at 0–40 μM Western blot analysis for PKM protein expression Glucose, lactate, and ATP assays	[100]
**Gliotoxin**	Brain cancer (glioblastoma) cells (U87, U251) Leukemia cells (HL-60, K-562) Non-small cell lung cancer cells (H1975) Prostate adenocarcinoma cells (PC-3) Colorectal cancer cells (HCT-116) Cervical adenocarcinoma cells (HeLa)	PK activity assay Cell viability assay (MTT assay, 72 h)—all cell lines Western blot assay (0.1–0.5, 48 h U87) Cellular thermal shift assay (CETSA assay 6 h, U87) Glucose and lactic acid assays (U87, U251)	[101]
**Pseurotin A**	Brain cancer (glioblastoma) cells (U87-MG, C6, U251, and SHG-44)	Cell growth inhibition assay (sulforhodamine B assay) Western blot assay for PKM protein expression	[102]
**Shikonin**	Breast adenocarcinoma cells (MCF7) Lung adenocarcinoma cells (A549) Cervical adenocarcinoma cells (HeLa)	Gel electrophoresis protein separation and MS detection PKM and LDH activity assay (20–200 μM and 40 μM) Glucose and lactate assays (2.5–20 μM) Cell viability assay (trypan blue assay, 6 h) at 10 μM	[103]
**Olive leaf extract enriched in oleuropein**	Melanoma cells (A375)	Cell viability assay (MTT assay, 24, 48, 72 h) at 0–800 µM Cell cycle analysis and wound healing assay RT-PCR and western blot for PKM2, GLUT1, and MCT4 expressions	[105]
Inhibitors of lactate dehydrogenase (LDH)			
**Galloflavin**	Hepatoma carcinoma cells (PLC/PRF/5)	Virtual screening (LDH-A) Assay on purified human LDH-A and LDH-B Cell proliferation assay (neutral red assay, 72 h) Mechanism of cell death (apotox-glotm Triplex Assay, 24 h) Inhibition of lactate production	[111]
**Epigallocatechin** **(*Spatholobus suberectus Dunn.* Subfractionation)**	Breast adenocarcinoma cells (MCF7, MDA-MB-231)	In vitro LDH-A activity assay (screening of subfractions) RT-PCR assay for LDH-A mRNA level Western blot assay for LDH-A protein expression In vivo tumor growth	[113]
**Wogonin**	Gastric cancer cells (SGC-7901) and lung adenocarcinoma cells (A549)	Cell viability assay (MTT assay, 48 h) at 5, 10, 15, 20, 25 and 30 µg/mL Trypan blue exclusion assay and cell morphological assessment (48 h, at 15 µg/mL) Enzyme activity assays (48 h at 15 µg/mL)	[108]
**(−)-epicatechin-3-O-gallate, ω-hydroxyemodin, emodin-1-O-gluciside, lunatin, chrysophanic acid, rhein-O-(acetyl)-glucoside, aloe-emodin, rhein and emodin**		LDH functionalized magnetic nanoparticles study	[114]

ATP—adenosine triphosphate; CE-MS—capillary electrophoresis mass spectrometry; G6PDH—glucose-6-phosphate -dehydrogenase; GLUT1—glucose transporter 1; GLUT3—glucose transporter 3; GLUT4—glucose transporter 4; HIF-1α—hypoxia-inducible factor 1-alpha; HK2—hexokinase 2; IC_50_—inhibitory concentration 50; IHC—Immunohistochemical; LDH—lactate dehydrogenase; LDH-A—lactate dehydrogenase A; LDH-B—lactate dehydrogenase B; MCT4—monocarboxylate transporter 4; mRNA-messenger ribonucleic acid; MS—mass spectrometry; MTT assay—2,5-diphenyl-2H-tetrazolium bromide-based assay; MTS assay—3-(4,5-dimethylthiazol-2-yl)-5-(3-carboxymethoxyphenyl)-2-(4-sulfophenyl)-2H-tetrazolium-based assay; NMR- Nuclear magnetic resonance spectroscopy; p53—tumor suppressor gene 53; p-Akt/Akt—phosphorylated protein kinase B/protein kinase B ratio; PCR—polymerase chain reaction; PDK—pyruvate dehydrogenase kinase; PFK—6-phosphofructo-2-kinase; PFKFB1/4—6-phosphofructo-2-kinase/fructose-2,6-biphosphatase1/4; PFKFB2—6-phosphofructo-2-kinase/fructose-2,6-biphosphatase 2; PFKL—ATP-dependent 6-phosphofructokinase liver type; PGM—phosphoglucomutase; PK—pyruvate kinase; PKM2—pyruvate kinase M2; p-mTOR/mTOR—phosphorylated mammalian target of rapamycin/mammalian target of rapamycin ratio; RT-PCR—real-time polymerase chain reaction; ROS—reactive oxygen species; STAT3—signal transducer and activator of transcription 3; TIGAR—TP53-induced glycolysis and apoptosis regulator; YAP—Yes-associated protein.

**Table 3:** Natural compounds were identified to negatively regulate the HIF—1α signaling pathway by different mechanisms.

Natural Compound	In Vitro Models	Study Design	Ref
Inhibitors of HIF-1α synthesis			
By targeting the upstream pathways			
**Apigenin**	Hepatocellular carcinoma cells (Hep G2)	0, 10, 20, and 40 μM for 12 h	[9]
**Biochanin A**	Glioblastoma multiforme cells (U251)	0, 50, and 100 μM for 48 h	[124]
**Berberine**	Colorectal carcinoma cells (HCT116, KM12C)	0–100 μM for 24 h for HCT116 cells or 15 h for KM12C cells	[125]
**Chrysin**	Prostate cancer cells (DU145)	serum-starved cells stimulated with insulin (200 nmol/L) for 6 h, followed by 30 μmol/L chrysin for 30 min	[143]
**Chlorogenic acid**	Lung cancer cells (A549)	2 μM or 10 μM for 16 h, followed by exposure to 200 μM cobalt chloride for 6 h	[144]
**Cryptotanshinone**	Bladder carcinoma cells (5637, T24)	0, 20, 40, and 80 μM for 48 h	[145]
**Epigallocatechin-3-gallate (EGCT) and green tea extract (GTE)**	Cervical adenocarcinoma cells (HeLa) and hepatocellular carcinoma (Hep G2) cells	10–80 μg/mL GTE, 10–100 μM EGCG, under hypoxic and normoxic conditions for 16 h	[146]
**Gambogic acid**	Multiple myeloma cells (U266)	0.2 μM for 4 h under hypoxic conditions	[147]
**Quercetin**	Colorectal carcinoma cells (HCT116), prostate cancer cells (DU145), cervical adenocarcinoma cells (HeLa S3)	0, 50, and 100 μM for 12 h	[136]
**Resveratrol**	Tongue squamous cell carcinoma (SCC-9) and hepatocellular carcinoma cells (Hep G2)	5, 50, and 100 μM under hypoxic and normoxic conditions for 1 h or 16 h	[148]
**Wogonin**	Colorectal carcinoma cells (HCT116)	20, 40, 60, 80, and 100 mM for 24 h	[149]
By direct mechanisms			
**Deguelin**	Non-small cell lung cancer cells (H1299, A549), prostate adenocarcinoma cells (PC-3), gastric cancer cells (MKN-45), breast adenocarcinoma cells (MCF7), renal carcinoma cells (786-0)	100 nM for 6 h, under hypoxic and normoxic conditions	[150]
**Dictamnine**	Colorectal carcinoma cells (HCT116), cervical adenocarcinoma cells (HeLa), hepatic adenocarcinoma cells (SK-Hep1), lung carcinoma cells (A549)	0, 10, 30, and 100 μM for 12 h, under hypoxic and normoxic conditions	[151]
**Epigallocatechin-3-gallate (EGCG)**	Pancreatic cancer cells (PANC-1)	0, 20, 40, and 80 µg/mL EGCG under hypoxic conditions for 24 h, no EGCG under normoxic conditions	[152]
**Genistein**	Breast cancer cells (MDA-MB-231, T-47D)	for MDA-MB-231 cells—100 µM for 24 h; for T-47D cells—50 µM for 24 h	[153]
Inhibitors of HIF-1α mRNA expression			
**Apigenin**	Pancreatic cancer cells (S2-013, CD18)	0-50 µM for 24 h, under hypoxic and normoxic conditions	[154]
**Curcumin**	Rodent (AtT20, GH3) and human pituitary tumor cells	0, 10, 20, and 30 μM for 30 min, followed by 125 or 250 μM cobalt chloride exposure for 3 h	[155]
	Papillary thyroid cancer cells (K1 PTC)	12.5, 25, and 50 mmol/L for 1 h, followed by exposure to hypoxia for an additional 12 h	[156]
Inhibitors of protein stabilization and accumulation			
**Apigenin**	Prostate adenocarcinoma cells (PC-3, DU145, and LNCaP), ovarian cancer cells (OVCAR-3), colon cancer cells (HCT-8), and breast adenocarcinoma cells (MCF7)	0, 20, and 40 μM for 1 h—depending on the experiment purpose	[8]
**Baicalein**	Breast adenocarcinoma cells (MCF7)	50 µM under hypoxic conditions or with 150 µM cobalt chloride for 8 h	[157]
**Chrysin**	Prostate cancer cells (DU145)	serum-starved cells stimulated with 200 nmol/L insulin for 6 h, followed by 30 μmol/L chrysin for 30 min	[143]
**Epigallocatechin-3-gallate (EGCG) and green tea extract (GTE)**	Cervical adenocarcinoma cells (HeLa) and hepatocellular carcinoma (Hep G2) cells	10–80 μg/mL GTE, 10–100 μM EGCG, under hypoxic and normoxic conditions for 16 h	[146]
**Kaempferol**	Hepatocellular carcinoma cells (Huh-7)	0, 1, 5, 10, and 50 μM for 4 h under hypoxic conditions (1% O_2_)	[138]
**Licochalcone A**	Colorectal carcinoma cells (HCT116), Non-small cell lung cancer cells (H1299), and bronchoalveolar carcinoma cells (H322)	For HCT116 cells: 5–20 μM, for 6 h (or 2–6 h) under hypoxic conditions	[158]
**Luteolin**	Colorectal carcinoma cells (HCT116), breast adenocarcinoma cells (MDA-MB-231)	0, 10, 25, and 50 μM for 48 h in the presence of 100 μM cobalt chloride for the last 24 h	[159]
**Magnolol**	Bladder cancer cells (T24)	0, 1, 5, and 10 µM for 8 h under normoxic or hypoxic conditions	[160]
**Oroxylin A**	Breast adenocarcinoma cells (MDA-MB-231)	50, 100, and 200 μM for 10 h, under hypoxic conditions	[161]
**Quercetin**	Prostate carcinoma cells (LNCaP), colon cancer cells (CX-1), and breast adenocarcinoma cells (SkBr3)	10–100 mM for 1, 2, 4, or 8 h, under normoxic or hypoxic conditions—depending on the experiment purpose	[162]
**Resveratrol**	Human osteosarcoma cells (Saos-2)	50 μM for 24 h	[163]
	Tongue squamous cell carcinoma (SCC-9) and hepatocellular carcinoma cells (Hep G2)	5, 50, 100 μM for 1 or 16 h, under hypoxic and normoxic conditions	[148]
**Wogonin**	Multiple myeloma cells (RPMI 8226, U266)	0, 20, 40, 80 μM for 24 h, under hypoxic and normoxic conditions	[164]
Inhibitors of transcriptional activity			
**Baicalein**	Breast adenocarcinoma cells (MCF7)	50 µM under hypoxic conditions or with 150 µM cobalt chloride for 8 h	[157]
**Chlorogenic acid**	Lung cancer cells (A549)	2, 10 μM for 16 h, followed by exposure to 200 μM cobalt chloride for 6 h	[144]
**Curcumin**	Hepatocellular carcinoma cells (Hep G2)	0, 25 μM, and 50 μM for 6 h under hypoxic conditions	[165]
**Genistein**	Hepatocellular carcinoma cells (HCC-LM3, SMMC-7721, Hep3B, BEL-7402, and Huh-7), normal hepatic cells (LO2)	for HCC-LM3 cells: 60 μM for 24 h	[166]
**Luteolin**	Colorectal carcinoma cells (HCT116), breast adenocarcinoma cells (MDA-MB-231)	0, 10, 25, and 50 μM for 48 h with 100 μM cobalt chloride for the last 24 h	[159]
**Quercetin**	Colorectal carcinoma cells (HCT116), prostate cancer cells (DU145), cervical adenocarcinoma cells (HeLa S3)	0, 50, and 100 μM for 12 h	[136]

## Data Availability

Data sharing not applicable.

## Supplementary Materials

The following supporting information can be downloaded at: www.mdpi.com/article/10.3390/ph15070808/s1, Table S1: Natural inhibitors of HIF-1α and their mechanisms of action, as identified in in vitro studies.
