# Supplementary material for: Phytochemicals as Regulators of Tumor Glycolysis and Hypoxia Signaling Pathways: Evidence from In Vitro Studies
Source: Pharmaceuticals (Basel). 2022 Jun 28;15(7):808. doi: 10.3390/ph15070808 (PMC9315613; doi:10.3390/ph15070808)
Supplement: Supplementary file 1 [file pharmaceuticals-15-00808-s001.zip › pharmaceuticals-1766135-supplementary.pdf]

pharmaceuticals

MDPI

Supplementary Table S1. Natural inhibitors of HIF-1α and their mechanisms of action, as identified in in vitro studies

Nr. Crt	Natural compound	In vitro model	Study design	Mechanism of action	Ref
1	Apigenin	Hepatocellular carcinoma cells (Hep G2)	0, 10, 20, and 40 µM for 12 h	- induction of apoptosis and autophagy through inhibition of PI3K/Akt/mTOR pathway	[9]
		Prostate adenocarcinoma cells (PC-3, DU145 and LNCaP), ovarian cancer cells (OVCAR-3), colon cancer cells (HCT-8), and breast adenocarcinoma cells (MCF7)	0, 20, and 40 µM for 1 h – depending on the experiment purpose	- suppression of tumor angiogenesis by inhibiting expression of HIF-1α and VEGF in tumor cells<br>- reduction of HIF-1α stability by blocking the binding of HIF-1α and Hsp90	[8]
		Pancreatic cancer cells (CD18, S2-013)	0–50 µM for 24 h, in normoxic and hypoxic conditions	- apigenin blocked hypoxia induced upregulation of HIF-1a, GLUT1, and VEGF proteins in both cell lines;	[154]
2	Baicalein	Hepatocellular carcinoma cells (Hep G2), neuroblastoma cells (SH-SY5Y), and mouse fibroblast cells (3T3-L1)	0–100 µM in normoxic or hypoxic (1% O_2) conditions, for different time sets (6 h, 16 h)	- abrogates asparaginyl hydroxylation of HIF-1α.<br>- suppresses ubiquitination of HIF-1α<br>- induces HIF-1-mediated reporter gene activity and target gene transcription<br>- induces HIF-independent activation of other genes.	[140]
		Breast adenocarcinoma cells (MCF7)	50 µM under hypoxic condition or with 150 µM cobalt chloride for 8 h	- inhibition of HIF-1α transcription activity<br>- inhibition of HIF-1α protein stabilization	[157]
3	Berberine	Colorectal carcinoma cells (HCT116, KM12C)	0–100 µM for 24 h for HCT116 cells or 15 h for KM12C cells	- inhibition of HIF-1α protein expression by suppression of mTOR pathway, interrupting the HIF-1α protein synthesis	[125]
4	Biochanin A	Glioblastoma multiforme cells	0, 50, and 100 µM for 48 h	- decreases the glycolytic capacity of the cells by	[124]

		(U251)		inhibiting the Akt/mTOR/HIF-1α signaling pathway	
5	Chlorogenic acid	Lung cancer cells (A549)	2 μM or 10 μM for 16 h, followed by exposure to 200 μM cobalt chloride for 6 h	- inhibition the HIF-1α/AKT signaling pathway<br>- suppresses the transcriptional activity of HIF-1α under hypoxic conditions	[144]
6	Chrysin	Prostate cancer cells (DU145)	serum-starved cells stimulated with 200 nmol/L insulin for 6 h, followed by 30 μmol/L chrysin for 30 min	- regulation of HIF-1α expression via PI3K/Akt pathway<br>- increases ubiquitination and degradation of HIF-1a by increasing prolyl hydroxylation<br>- interferes with interaction between HIF-1α and heat shock protein 90	[143]
7	Cryptotanshinone	Bladder carcinoma cells (5637, T24)	0, 20, 40, and 80 μM for 48 h	- suppresses proliferation and invasion by inhibiting PI3K/Akt pathway via PTEN activation	[145]
8	Curcumin	Rodent (AtT20, GH3) and human pituitary tumour cells	0, 10, 20, and 30 μM for 30 min, followed by 125 or 250 μM cobalt chloride exposure for 3 h	- blocked hypoxia-induced mRNA synthesis and secretion of VEGFA	[155]
		Hepatocellular carcinoma cells (Hep G2)	0, 25 μM, and 50 μM for 6 h under hypoxic conditions	- suppression of the HIF-1 transcriptional activity under hypoxia, leading to a decrease in the VEGF expression	[165]
		Papillary thyroid cancer cells (K1 PTC)	12.5, 25, and 50 mmol/L for 1 h, followed by exposure to hypoxia for an additional 12 h	- dose-dependent inhibition of the HIF-1α mRNA expression<br>- upregulation of HIF-1α protein expression<br>- decrease of DNA-binding activity of HIF-1α	[156]
9	Deguelin	Non-small cell lung cancer cells (H1299, A549), prostate	100 nM for 6 h under hypoxic and normoxic conditions	- inhibition of HIF-1α expression by inhibiting *de novo*	[150]

		adenocarcinoma cells (PC-3), gastric cancer cells (MKN-45), breast adenocarcinoma cells (MCF7), renal carcinoma cells (786-0)		protein synthesis and by inducing ubiquitin- and proteasome-mediated protein degradation	
10	Dictamnine	Colorectal carcinoma cells (HCT116), cervical adenocarcinoma cells (HeLa), hepatic adenocarcinoma cells (SK-Hep1), lung carcinoma cells (A549)	0, 10, 30, and 100 μM for 12 h, under hypoxic and normoxic conditions	- promotes apoptosis and inhibits epithelial-mesenchymal transition, migration, invasion, and proliferation by downregulating the HIF-1α and Slug signaling pathways<br>- inhibition of HIF-1α protein synthesis, but not degradation	[151]
11	Epigallocatechin-3-gallate (EGCG)	Pancreatic cancer cells (PANC-1)	0, 20, 40, and 80 μg/mL EGCG in hypoxic conditions for 24 h, no EGCG under normoxic conditions	- inhibition of HIF-1α protein expression in a dose-dependent manner<br>- no effects on HIF-1α mRNA expression	[152]
12	EGCG and green tea extract (GTE)	Cervical adenocarcinoma cells (HeLa) and hepatocellular carcinoma cells (Hep G2)	10–80 μg/mL GTE, 10–100 μM EGCG, in normal culturing conditions followed by exposure to normoxia or hypoxia for 16 h	- inhibition of hypoxia- and serum-induced HIF-1α protein accumulation by blocking both PI3K/Akt and ERK1/2 signaling pathways and enhancing of HIF-1a protein degradation through the proteasome system<br>- no effects on HIF-1α mRNA expression	[146]
13	Galangin	Ovarian cancer cells (OVCAR-3, A2780/CP70)	0, 10, 20, and 40 μM	- decrease the levels of HIF-1α, phosphor-Akt, phosphor-P70S6K proteins<br>- no effect on NFκB and PTEN	[141]
14	Gambogic acid	Multiple myeloma cells (U266)	0.2 μM for 4 h under hypoxia	- reduction Akt and mTOR phosphorylation	[147]
15	Genistein	Breast cancer cells (MDA-MB-231, T-47D)	for MDA-MB-231 cells—100 μM, 24 h; for	- directly binds to HIF-1α and reduces HIF-1α expression	[153]

			T-47D cells—50 μM, 24 h		
		Hepatocellular carcinoma cells (HCC-LM3, SMMC-7721, Hep3B, BEL-7402, and Huh-7), normal hepatic cells (LO2)	HCC-LM3 cells: 60 μM for 24 h	- downregulation of HIF-1α, which led to GLUT1 and HK2 inactivation and aerobic glycolysis suppression<br>- inhibition of protein expression and transcription activity of HIF-1α in a dose-dependent manner	[166]
16	Kaempferol	Hepatocellular carcinoma cells (Huh-7)	0, 1, 5, 10, and 50 μM for 4 h under hypoxic conditions (1% O_2)	- inhibition of HIF-1α activity and nuclear accumulation<br>- inactivation of p44/42 MAPK pathway	[138]
17	Licochalcone A	Colorectal carcinoma cells (HCT116), Non-small cell lung cancer cells (H1299) and bronchioalveolar carcinoma cells (H322)	HCT116 cells: incubation for 6 h (or 2–6 h) in the presence or absence of 5 - 20 μM licochalcone A under hypoxic conditions.	- inhibition of HIF-1α accumulation and expression of its target genes<br>- inhibition of mitochondria respiration and increase in intracellular oxygen	[158]
18	Luteolin	Colorectal carcinoma cells (HCT116), breast adenocarcinoma cells (MDA-MB-231)	0, 10, 25, and 50 μM for 48 h in the presence of 100 μM cobalt chloride the last 24 h	- reduction of nuclear HIF-1α accumulation in HCT116 cells<br>- inhibition of HIF-1-dependent transcriptional activity induced by cobalt chloride	[159]
19	Magnolol	Bladder cancer cells (T24)	0, 1, 5, and 10 μM for 8 h under normoxic or hypoxic conditions	- enhances prolyl hydroxylase activity and reduces HIF-1α protein accumulation	[160]
20	Myricitin	Ovarian cancer cells (OVCAR-3, A2780/CP70)	0, 5, 10, and 20 μM	- decrease of HIF-1α, phosphor-Akt, phosphor-P70S6K protein levels<br>- no effect on NFκB and PTEN	[141]
21	Oridonin	Gallbladder carcinoma cells (GBC-SD)	5 μM for 6, 12, and 24 h	- inhibition of hypoxia-induced epithelial–mesenchymal transition and cell migration by downregulating the expression levels of (HIF)-1α/matrix metallopeptidase 9	[142]

				(MMP9)	
22	Oroxylin A	Breast adenocarcinoma cells (MDA-MB-231)	50, 100, and 200 μM for 10 h, under hypoxic conditions	- inhibition of glycolysis-dependent proliferation through the suppression of HIF-1α stabilization via SIRT3 activation	[161]
23	Quercetin	Prostate carcinoma cells (LNCaP), colon cancer cells (CX-1) and breast adenocarcinoma cells (SkBr3)	10–100 mM for 1, 2, 4, or 8 h, under normoxic or hypoxic conditions – depending on the experiment purpose	- inhibition of HIF-1α accumulation through inhibition of protein synthesis under hypoxic conditions	[162]
		Colorectal carcinoma cells (HCT116), prostate cancer cells (DU145), cervical adenocarcinoma cells (HeLa S3)	0, 50, and 100 μM for 12 h	- inhibition of AMPK activity under hypoxic conditions, leading to reduction of HIF-1 activity<br>- reduction of hypoxia-induced transcriptional activity of HIF-1	[136]
24	Resveratrol	Human osteosarcoma cells (Saos-2)	50 μM for 24 h	- inhibition of HIF-1α protein accumulation and promotion of HIF-1α protein degradation, without affecting the HIF-1α mRNA level	[163]
		Human tongue squamous cell carcinoma (SCC-9) and human hepatocellular carcinoma (Hep G2) cells	5, 50, and 100 μM for 1 h or 16 h, under hypoxic and normoxic conditions	- inhibition of hypoxia-induced HIF-1α protein accumulation and VEGF expression through the inhibition of PI-3K/Akt and p42/p44 MAPK activation	[148]
25	Wogonin	Colorectal carcinoma cells (HCT116)	20, 40, 60, 80, and 100 mM for 24 h	- reverses hypoxia resistance via downregulation of HIF-1α and glycolysis, by inhibiting PI3K/Akt signaling pathway	[149]
		Multiple myeloma cells (RPMI 8226, U266)	0, 20, 40, and 80 μM for 24 h under normoxic and	- inhibition of c-Myc expression and promotion of HIF-1α degradation	[164]

hypoxic conditions
